# Identification of Quiescent, Stem-Like Cells in the Distal Female Reproductive Tract

**DOI:** 10.1371/journal.pone.0040691

**Published:** 2012-07-24

**Authors:** Yongyi Wang, Andrea Sacchetti, Matthijs R. van Dijk, Marten van der Zee, Paul H. van der Horst, Rosalie Joosten, Curt W. Burger, J. Anton Grootegoed, Leen J. Blok, Riccardo Fodde

**Affiliations:** 1 Departments of Obstetrics & Gynecology, Josephine Nefkens Institute, Erasmus MC, Rotterdam, The Netherlands; 2 Department of Pathology, Josephine Nefkens Institute, Erasmus MC, Rotterdam, The Netherlands; 3 Department of Reproduction and Development, Erasmus MC, Rotterdam, The Netherlands; Baylor College of Medicine, United States of America

## Abstract

In fertile women, the endometrium undergoes regular cycles of tissue build-up and regression. It is likely that uterine stem cells are involved in this remarkable turn over. The main goal of our current investigations was to identify slow-cycling (quiescent) endometrial stem cells by means of a pulse-chase approach to selectively earmark, prospectively isolate, and characterize label-retaining cells (LRCs). To this aim, transgenic mice expressing histone2B-GFP (H2B-GFP) in a Tet-inducible fashion were administered doxycycline (pulse) which was thereafter withdrawn from the drinking water (chase). Over time, dividing cells progressively loose GFP signal whereas infrequently dividing cells retain H2B-GFP expression. We evaluated H2B-GFP retaining cells at different chase time points and identified long-term (LT; >12 weeks) LRCs. The LT-LRCs are negative for estrogen receptor-α and express low levels of progesterone receptors. LRCs sorted by FACS are able to form spheroids capable of self-renewal and differentiation. Upon serum stimulation spheroid cells are induced to differentiate and form glandular structures which express markers of mature Műllerian epithelial cells. Overall, the results indicate that quiescent cells located in the distal oviduct have stem-like properties and can differentiate into distinct cell lineages specific of endometrium, proximal and distal oviduct. Future lineage-tracing studies will elucidate the role played by these cells in homeostasis, tissue injury and cancer of the female reproductive tract in the mouse and eventually in man.

## Introduction

Stem cells are relatively undifferentiated and naive cells endorsed with the ability to self-renew and to give rise to committed progenitors and differentiated cell lineages. Somatic stem cell niches such as skin [1], stomach [2], [3], intestine [4], [5], [6], [7] and bone marrow [8] have been shown to encompass both quiescent and cycling populations. Whereas cycling stem cells maintain daily homeostasis, their quiescent equivalents have been postulated to play a rate-limiting role in tissue regeneration upon injury [9], [10]. To date, very little is known about the nature and localization of stem cells in the female reproductive tract and in particular in the uterus [11].

The very first evidence for the existence of a stem cell population in the endometrium came by assaying the clonogenicity of single endometrial cells *in vitro*. It was observed that 0.08% of epithelial and 0.02% of stromal cells formed large colonies which could be passaged several times [12]. Furthermore, passaging of epithelial colonies in matrigel resulted in large cytokeratin expressing gland-like structures [13]. Additional studies on the identification of endometrial stem cells indicated that, in bone-marrow transplanted patients, donor-derived bone marrow cells could be found in the endometrial epithelium and stroma [14], [15].

Bromodeoxyuridine (BrdU) label retention was also employed to identify slow cycling cells in the endometrium, a feature of quiescent stem cells: after 8 weeks of chase approximately 3% of endometrial epithelial cells still retained the BrdU label [16]. After 12 weeks of chase, no label-retaining cells (LRCs) were present in the luminal and glandular epithelium whereas few stromal BrdU-positive cells were observed at the endometrial-myometrial junction in close proximity to the blood vessels. However, DNA-labeling by BrdU is dependent on cell division and in itself represents a genotoxic insult for quiescent stem cells which may trigger their cell cycle activation and the progressive dilution of the label [8].

Here, we have employed a non-mutagenic and cell cycle independent approach, namely *in vivo* pulse-chase with the histone 2B – green fluorescent protein (H2B-GFP) [1], [8], [17], towards the identification and prospective isolation of long-term label-retaining cells (LT-LRCs) in the mouse female reproductive tract. To this aim, we have bred a transgenic model expressing the reverse transactivator rtTA2S-M2 under the control of the ubiquitous and methylation-free CpG island of the human hnRNPA2B1-CBX3 gene [18] with the tetO-HIST1H2BJ/GFP (H2B-GFP) mice [1]. In this way, upon doxycycline administration in the drinking water (pulse), the H2B-GFP marker protein is expressed in ubiquitous fashion. Upon doxycycline withdrawal (chase), actively cycling cells progressively dilute the nuclear H2B-GFP whereas infrequently dividing and quiescent cells will retain the label for longer intervals of time.

We show that LT-LRCs persist in the distal oviduct for up to 47 weeks of chase and that *ex-vivo* culture of these cells gives rise to undifferentiated spheroids which display self-renewal capacity and can be induced to differentiate into cells resembling different derivatives of the female embryonic reproductive tract, the Müllerian duct.

## Results and Discussion

### Identification and characterization of LT-LRCs in the distal oviduct

**Figure 1:**
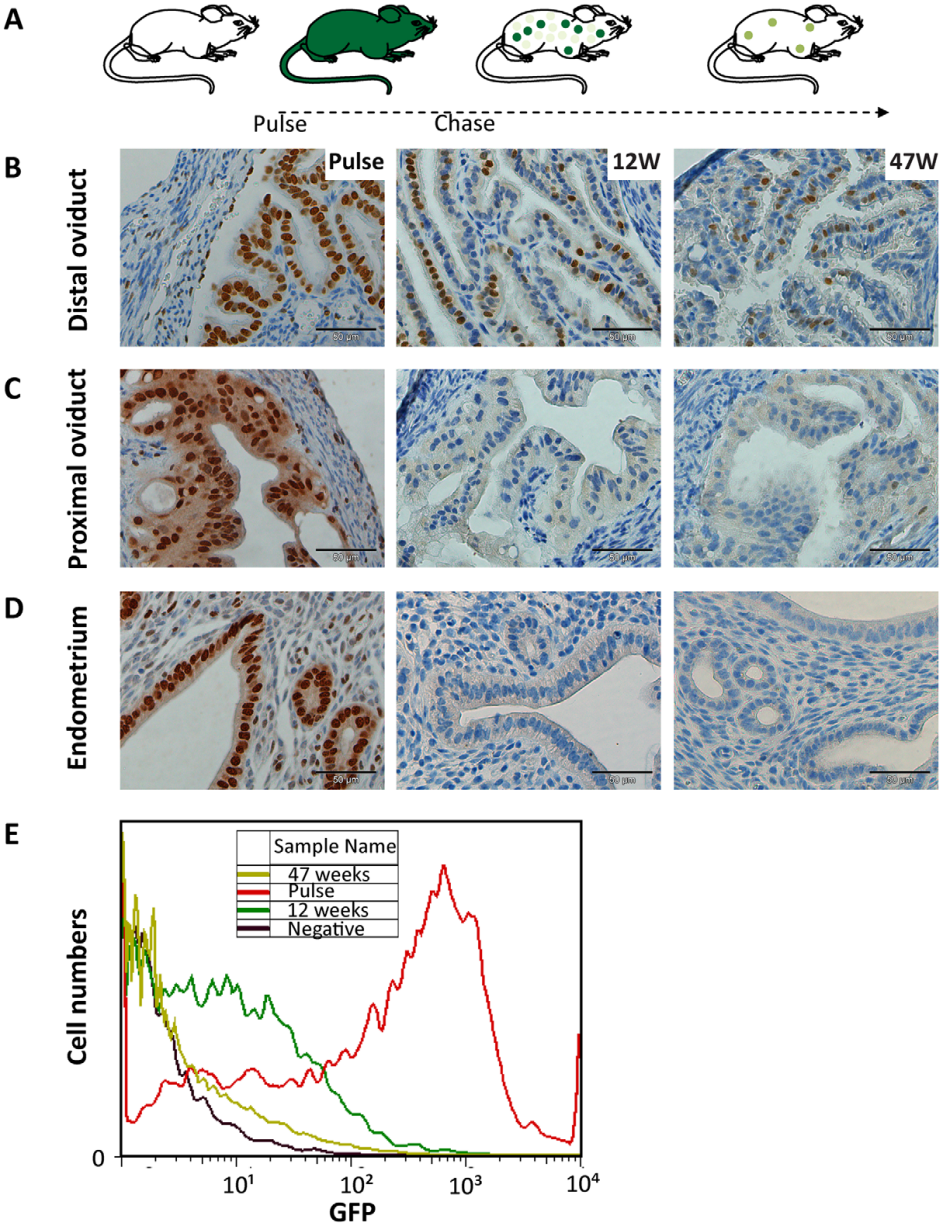
Pulse-chase experiment using the doxycycline-inducible H2B-GFP system. A schematic representation of the experiment is given in A, where a 7-day treatment with doxycycline (pulse) results in expression of H2B-GFP throughout the entire organism. At different chase time points (12 and 47 weeks after pulse), the GFP signal is progressively diluted from dividing cells and retained in quiescent cells in the distal oviduct (B). In the proximal oviduct (C) and the endometrium (D), label-retaining cells (LRCs) are lost at both 12 and 47 weeks chase time points. FACSorting was performed on single cell digestions of oviducts from different mice. In (E), the GFP signal is plotted against the number of cells. Animals used were: untreated mice as negative controls (black line, Negative); mice pulsed for 7 days (no chase) as positive controls (red line, Pulse); mice pulsed for 7 days and chased for 12 weeks (green line, 12 weeks); and mice pulsed for 7 days and chased for 47 weeks (light-green line, 47 weeks). The scale bar represents 50 μM.

H2B-GFP labeling of the vast majority of uterine cells was observed after 7 days of doxycycline pulse both by immunohistochemistry (IHC; Figure 1) and immunofluorescence (Figure S1A). Notably, the H2B signal appeared much higher in epithelial than in stromal or myometrial cells. In Figure 1, IHC analysis of H2B-GFP after 7 days of doxycycline treatment showed clear and complete epithelial staining in the distal and proximal oviduct, and in the endometrium (Figure 1B–D; left panels). Upon doxycycline withdrawal (chase), it is expected that dividing cells progressively lose their H2B-GFP signal, while quiescent or infrequently dividing cells will retain the label for longer chase periods (Figure 1A). In the endometrium, epithelial cells appeared to completely lose H2B-GFP expression within 2 to 4 weeks, whereas stromal LRCs lost H2B-GFP expression between 8 and 12 weeks of chase (Figure S1). These results are largely in agreement with those by Chan et al. [16] although in our pulse-chase analysis the glandular epithelium appeared to loose its label at a slower rate than the luminal epithelium [16]. In the proximal oviduct no label retaining cells were observed after 12 weeks of chase (Figure 1C). Remarkably however, many LRCs were found after 12 weeks of chase in the distal oviduct (Figure 1B, Figure S2). Furthermore, after an extensive 47 week chase multiple LRCs are still present in the distal oviduct (Figure 1B). Here, we will refer to LRCs persisting for at least 12 weeks of chase and onwards, as long-term label-retaining cells (LT-LRCs).

The above results were confirmed by FACS sorting of GFP^+^ cells from whole oviducts before pulse (untreated animals as negative controls) and at chase times 0, 12 and 47 weeks (Figure 1E). Oviductal cells from untreated mice showed a very low GFP signal (black line). In contrast, after 1 week of pulse (chase time 0 weeks, red line) a large portion of cells displayed a strong GFP signal. At increasing chase times, oviductal cells show substantial loss of their GFP signal (dark-green line; chase time 12 weeks, 11% GFP^+^ cells), whereas after 47 weeks of chase only very few GFP^+^ cells are present (Figure 1E, light-green line, 0.5% GFP^+^ cells). Based on the above, all subsequent analyses were performed at 12 weeks of chase.

**Figure 2:**
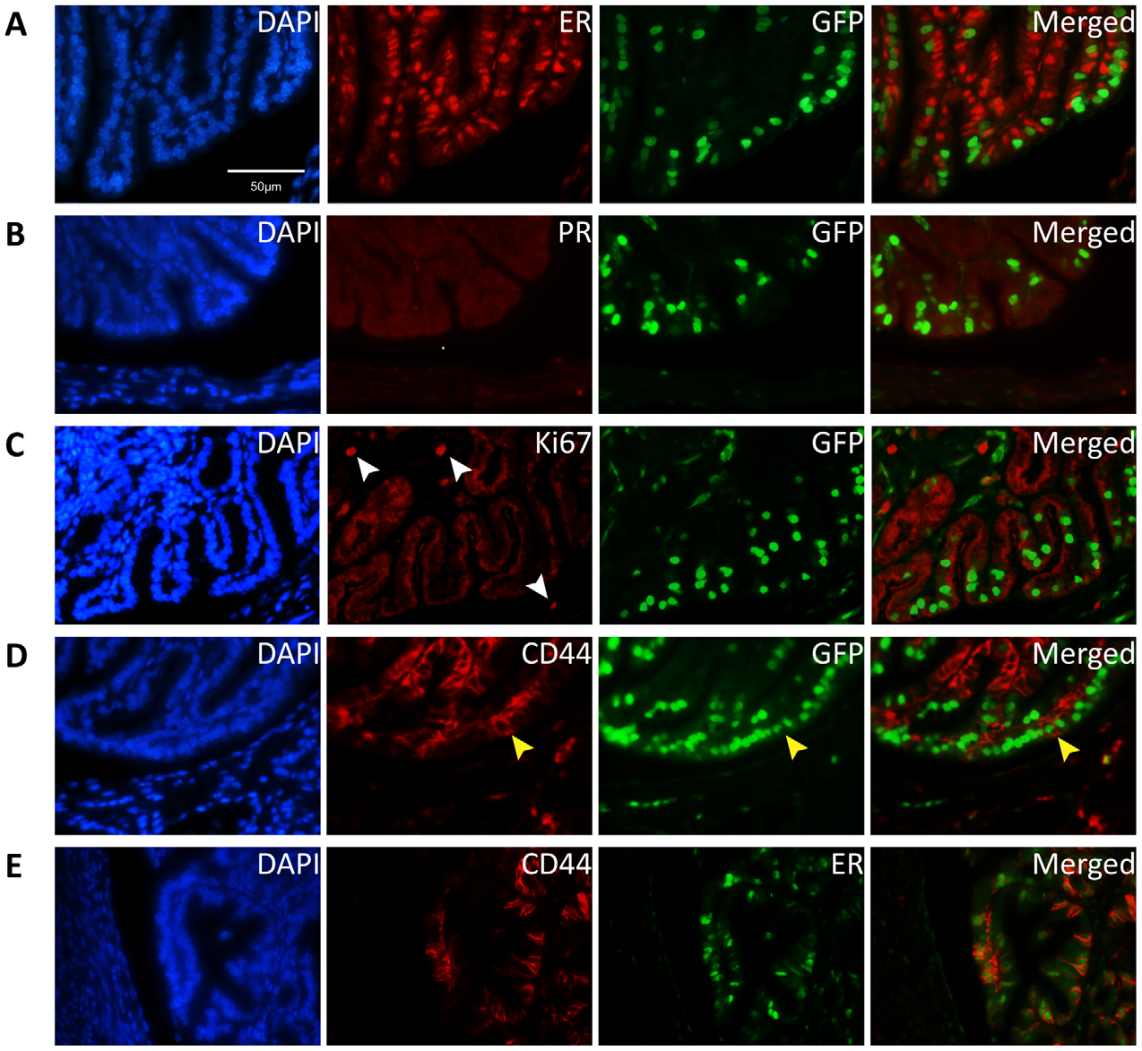
Characterization of identified LT-LRCs (**12 week chase**) **in mouse distal oviduct.** Immunofluorescent double-labeling was performed for GFP and ERα (A), GFP and PR (B), GFP and Ki67 (C), GFP and CD44 (D) and CD44 and ERα (E). DAPI staining was performed to display all cell nuclei. White arrowheads indicate Ki67 positive cells, the yellow arrowhead indicates a cell which is positive for CD44 as well as GFP.

To characterize the newly identified LT-LRCs in the distal oviduct, immunofluorescent staining analysis was performed for expression of estrogen and progesterone receptors, CD44 as a stem cell marker in the mammary and other epithelial niches [19], [20], and Ki67 as an indicator of proliferative activity. In the mammary gland, epithelial cells are known to be both estrogen and progesterone receptor positive, yet the stem cells in that tissue do not express either receptors [21], [22], [23]. Furthermore, the vast majority of the epithelial cells from the female reproductive tract are known to response to estrogenic and progestagenic hormones [24]. Expression of estrogen receptor alpha (ERα) and progesterone receptors A and B (PR) was assessed in LT-LRCs (GFP^+^) after 12 weeks of chase. As shown in Figure 2A, LT-LRCs were found not to express ERα. Furthermore, in contrast to the endometrium, the distal oviduct displayed very low levels of PR expression (Figure 2B and Figure S3) and, accordingly, we conclude that LT-LRCs appear negative for PR expression (Figure 2B). To validate the quiescent nature of LT-LRCs, expression of the proliferation marker Ki67 was also evaluated. Overall, very few Ki67-positive cells were found in the distal oviduct and, accordingly, double positive cells for GFP and Ki67 were never observed (Figure 2C and Figure S4). As for CD44, many cells throughout the distal oviduct were positive, though seldom in GFP^+^ cells (Figure 2D). Notably, double staining analysis revealed that most CD44-expressing cells also expressed ERα (Figure 2E). Furthermore, upon staining for GFP and putative stem cell markers Sca1, Msi1, Lgr5, and c-Kit, no double staining was observed (data not shown).

Overall, these data indicate that a cluster of epithelial LT-LRCs persist within the distal oviduct for up to 47 weeks after the pulse, whereas other segments of the female reproductive tract seem to be characterized by a considerably higher turnover. Interestingly, during embryonic development of the Müllerian duct, the anterior region of the coelomic cavity, where mesoepithelial cells are induced to invaginate by Wnt4 expressing cells from the Wolffian duct, gives rise in a later stage to the distal oviduct. This initial invagination elongates alongside the Wolffian duct under the influence of Wnt9b [25] and a population of rapidly proliferating progenitor cells at the tip of the elongation is responsible for the outgrowth which will eventually form the Müllerian ducts [26].

### Ex-vivo isolated long-term LRCs can form epithelial spheroids

**Figure 3:**
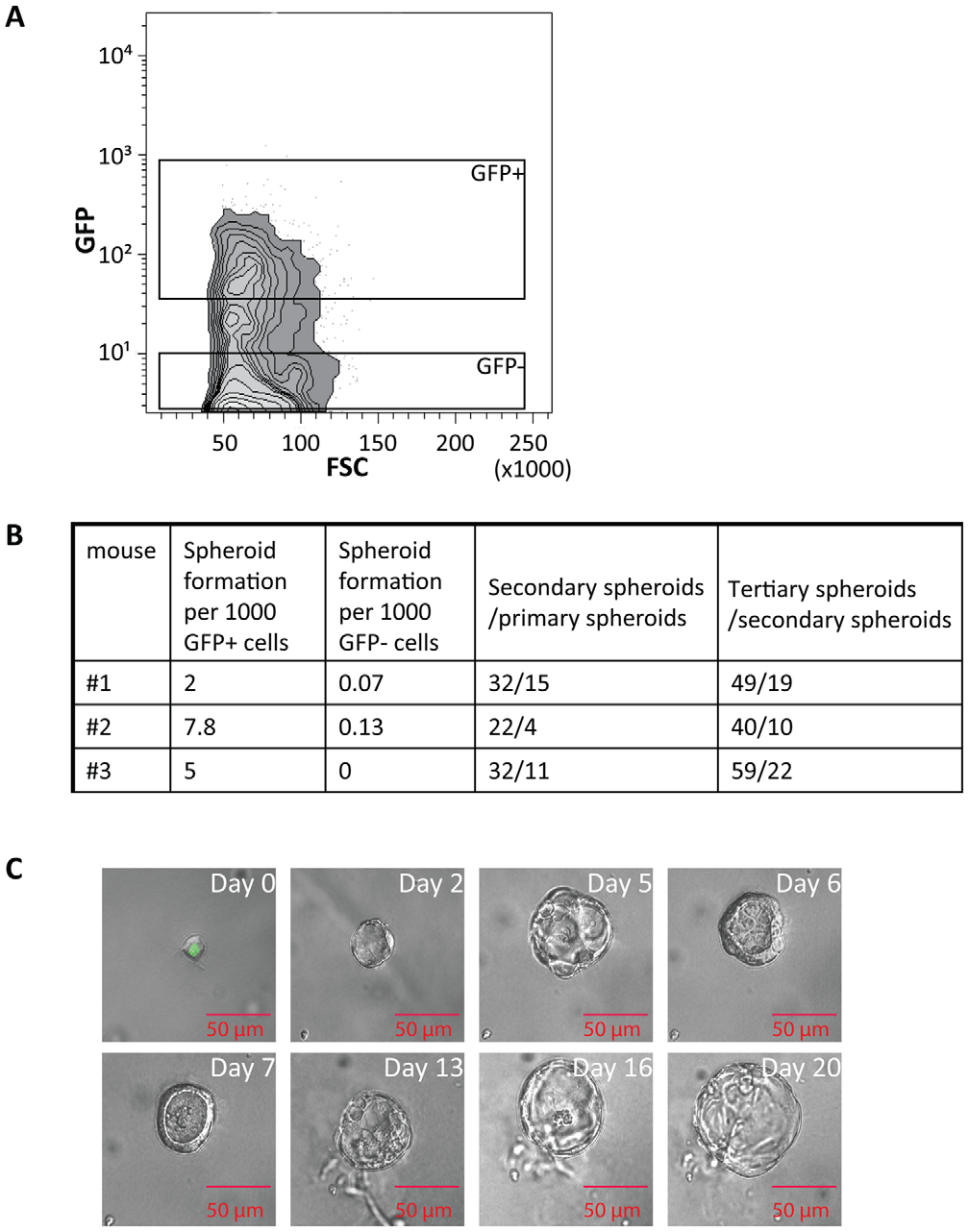
Isolated LT-LRC can form self-renewing spheroids in culture. Oviducts from 12 week chased mice were dissected and a single cell suspension was analyzed by FACS. From three different 12 week chased mice, GFP^+^ and GFP-negative (GFP−) cells were isolated by FACSorting and cultured in Matrigel under serum-free conditions to assay their capacity to form spheroids (A). Self-renewal was assayed by dissociating spheroids and plating the unsorted cell suspension to form new spheroids as indicated in the Materials and Methods section. In short, primary spheroids (i.e. obtained directly from the chased animals) were reduced to single cell suspensions and plated again (unsorted) in matrigel/serum-free culture conditions. The corresponding secondary organoids were then dissociated and plated to obtain tertiary ones (B). Using limiting dilutions, individual GFP^+^ cells were cultured for 0 to 20 days in Matrigel under serum-free conditions, thus forming a polarized epithelial organoid. The bars represent 50 μM (C).

When cultured in serum free and in 3D conditions (e.g. matrigel), stem cells are able to form organoid structures often referred to as spheroids. In order to provide indication of the presence of stem-like cells among the LT-LRCs isolated *ex-vivo* from the distal oviduct, we sorted GFP^+^ and GFP-negative cells by FACS from the oviducts of 12-week chased animals (Figure 3A). Sorted single cells were subsequently cultured in matrigel under serum-free conditions to assay their capacity to form spheroids. As shown in Figure 3B, GFP+ cells form spheroids at a significantly higher rate (average of mouse 1, 2 and 3 =  ∼1 spheroid/200 cells) than GFP-negative cells (average of mouse 1, 2 and 3 =  ∼1 spheroid/15000 cells). Furthermore, spheroids obtained from GFP-negative cells maintained significantly smaller (and survived only for short-term cultures) than spheroids formed from GFP+ cells. The observation according to which only a minority (1/200) of the GFP+ cells is capable of forming spheroids may have different explanations. First, the LRCs encompass a heterogeneous group of post mitotic, terminally differentiated cells together with quiescent stem-like cells. Second, the experimental procedure is likely to negatively affect the organoid-forming capacity of the sorted cells. Last but not least, being intrinsically quiescent, the LRCs are likely to need specific stress-stimuli to be optimally activated to enter the cell cycle and eventually form organoids in matrigel culture assays.

Next, enzymatic and mechanic dissociation of these primary organoids was applied to obtain single cells to be plated in matrigel and serum-free culture conditions. Secondary spheroids were exclusively obtained from cells isolated from primary spheroids derived from GFP^+^ cells and not from those obtained from GFP-negative cells. Likewise, tertiary spheroids were obtained from the secondary organoids (Figure 3B).

Next, we followed single LT-LRCs during the spheroid-formation process. Using limiting dilutions, individual GFP^+^ cells were first recognized and observed to divide and progressively form an unstructured organoid between days 2 and 5 (Figure 3C). Over the course of the next few days (day 6–20), early organoids kept growing, simultaneously forming a polarized epithelial-like outer layer of the spheroid (Figure 3C). In order to confirm the epithelial mature spheroids were also stained for cytokeratin 8 (CK8) and membrane β-catenin expression (Figure S5).

**Figure 4:**
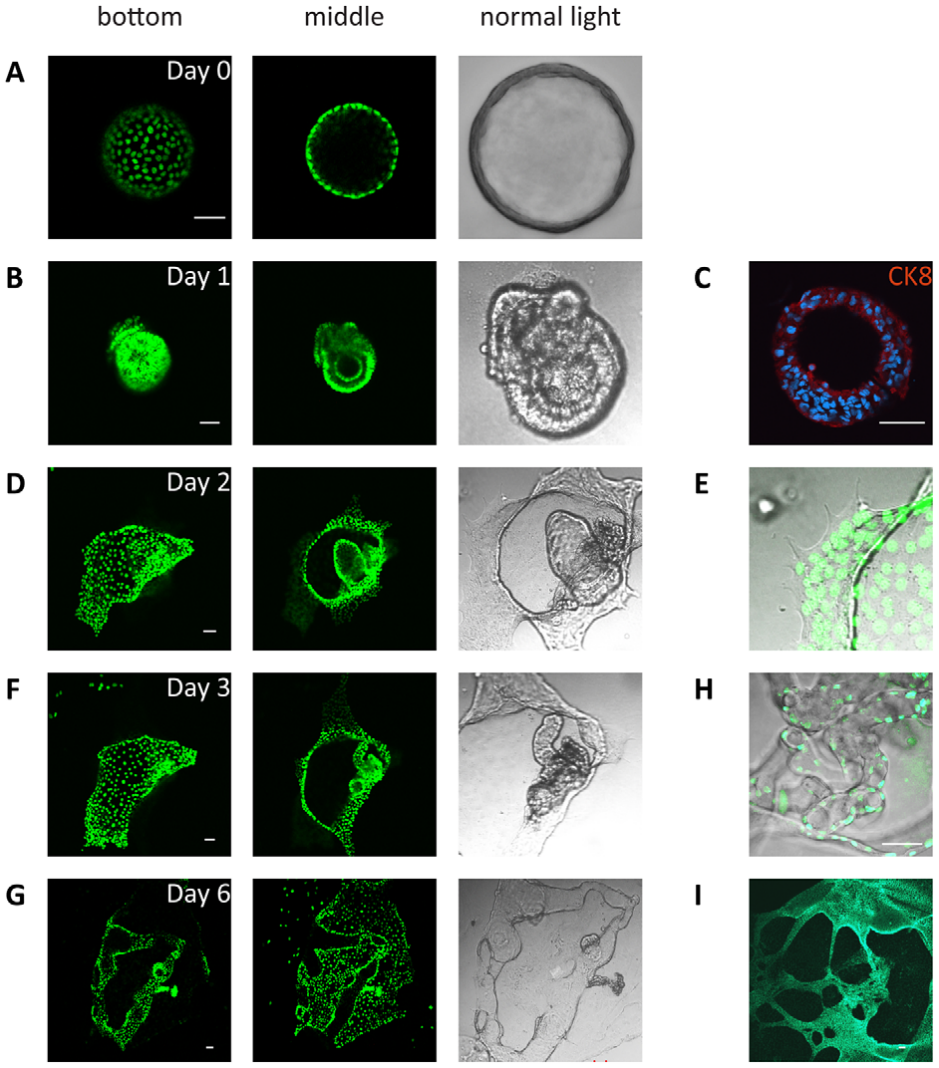
Spheroids differentiation assay. 20 day old spheroids derived from oviducts of 12 week chased mice were pulsed for one day with doxycycline and were subsequently allowed to differentiated for 0 (A), 1 (B and C), 2 (D and E), 3 (F) or 6 days (G) in the presence of 10% FCS. The two columns of images on the left hand side of the figure represent confocal images taken at two different planes (bottom and middle). The phase-contrast images in the third colum from the left represents a detail from the confocal image. C represents a spheroid derived from a different animal which was induced to differentiate for 1 day, displaying formation of multiple cell layers as indicated by nuclear DAPI staining and immunofluorescence for CK8. In E attachment of the spheroid to the culture dish is shown. H and I represent spheroids isolated from different animals that were allowed to differentiate for 9 days (H) and for 20 days (I) showing complex structures (nuclei were stained with Hoechst 34580).

Under serum-free conditions, spheroids continue to grow slowly but steadily and maintain an undifferentiated appearance for at least 10 weeks. However, upon exposure to serum-supplemented (10% v/v FCS) medium, cell morphology undergoes dramatic modifications (Figure 4). In order to facilitate the visualization of cell morphology during the differentiation process, spheroids were cultured in the presence of doxycycline to re-induce H2B-GFP expression, and followed by using confocal and phase contrast microscopy and 3D image reconstruction (Figure 4 and Movie S1). Already one day after serum exposure, the formation of multiple cell layers was observed (Figure 4B and 4C). At day two, monolayers of organoid-derived cells were observed to spread out of spheroids which came in contact with the plastic surface of the culture dish (Figure 4D and 4E). Furthermore, additional small hollow structures appeared to bud out of the differentiating organoids (Figure 4D). At day 3, the budded structures appeared as a hollow tube occasionally flanked by secondary budding structures (Figure 4F). Subsequently, cells continued to divide, as shown by loss of GFP signaling (after doxycycline withdrawal from the culture medium), with tube elongation proceeding (Figure 4G). When, in independent experiments, spheroids were allowed to differentiate for 9 (Figure 4H) and 20 days (Figure 4I) respectively, formation of more complex structures was observed.

In summary, LT-LRCs isolated *ex-vivo* from the distal oviduct were able to form undifferentiated, self-renewing spheroids which, upon differentiation, gave rise to epithelial structures with a somewhat higher level of complexity.

**Figure 5:**
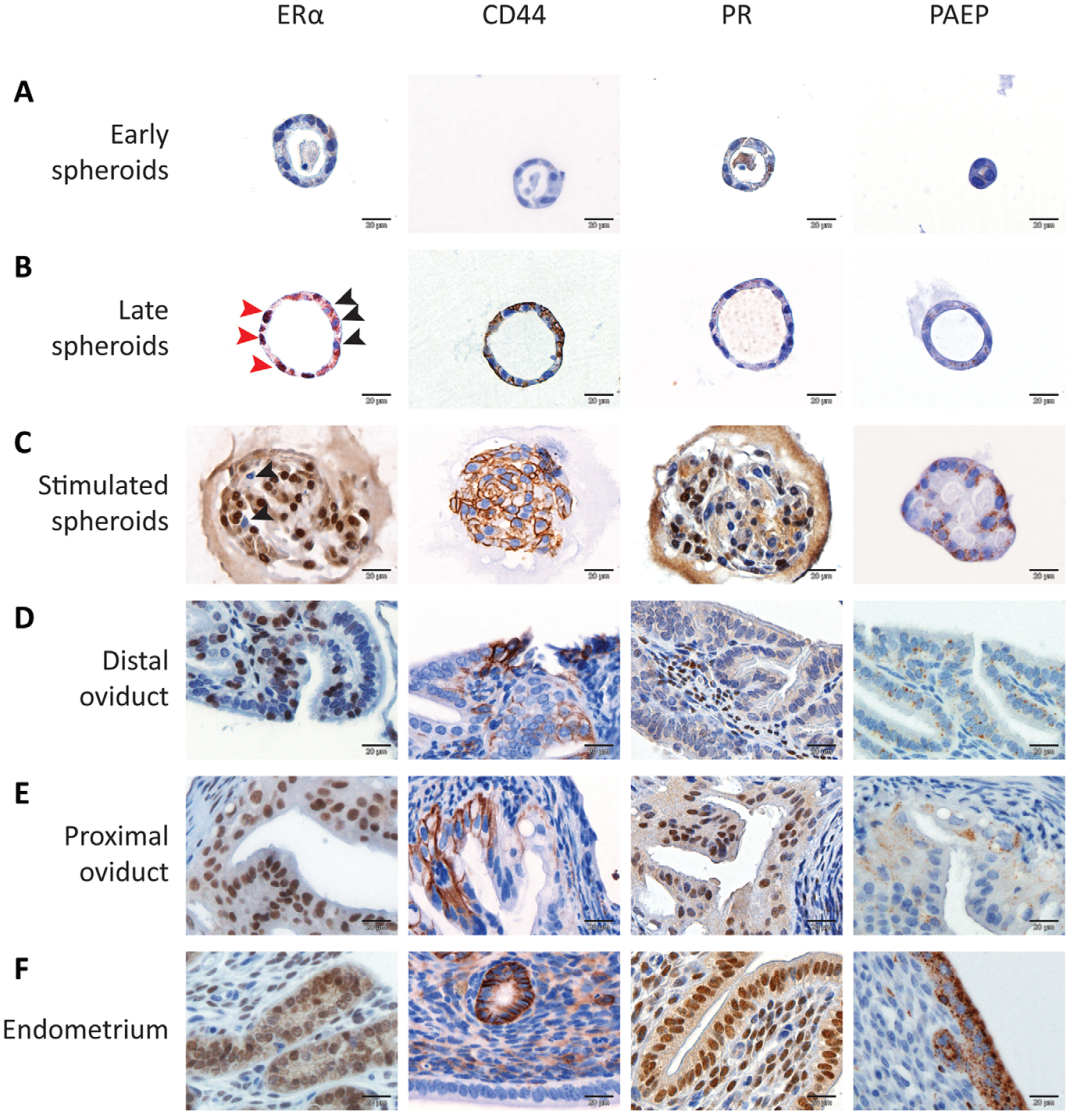
Differentiation of spheroids towards specific cell lineages of the female reproductive tract. Early spheroids (2 days of culture, A), late spheroids (2 weeks of culture, B) and spheroids which were stimulated to differentiate (10% FCS added to the culture medium for 2 days, C) were stained for ERα, CD44, PR and PAEP expression and compared to stained sections from mice distal oviduct (D), proximal oviduct (E) and endometrium (F). The red arrows indicate ERα positive cells, the black arrows indicate ERα negative cells. In panel A, 4 different spheroids were used, in panel B two (ER and PR, CD44 and PEAP stainings were performed on the same spheroid, respectively) and stainings in panel C are on consecutive sections from one spheroid. Images are representative for three different experiments containing more than 10 early and late spheroids and 2 – 3 differentiated spheroids.

To assess the capacity of the LT-LRC-derived organoids to differentiate towards specific cell lineages of the female reproductive tract, early (1 or 2 days culture), late (2 weeks culture), and more differentiated (see above) spheroids were analyzed by IHC for expression of ERα, CD44, PR and PAEP (progestagen-associated endometrial protein; glycodelin A) (Figure 5). Whereas in early spheroids all cells were negative for ERα, CD44, PR and PAEP (Figure 5A), late spheroids showed a distinct expression pattern with increased ERα and CD44 expression though ERα negative cells could still be recognized (Figure 5B, black arrow). PR and PAEP expression was negative (Figure 5B). Upon staining of the distal oviduct for the same markers (Figure 5D) it was observed that the expression pattern of distal oviduct overlapped with that of the late spheroids. Further induction of spheroid differentiation resulted in most cells expressing ERα, PR, CD44, and PAEP (Figure 5C). Notably, comparison of the expression data between differentiated spheroids and the proximal oviduct and endometrium (Figure 5E and 5F) revealed marked similarities.

**Figure 6:**
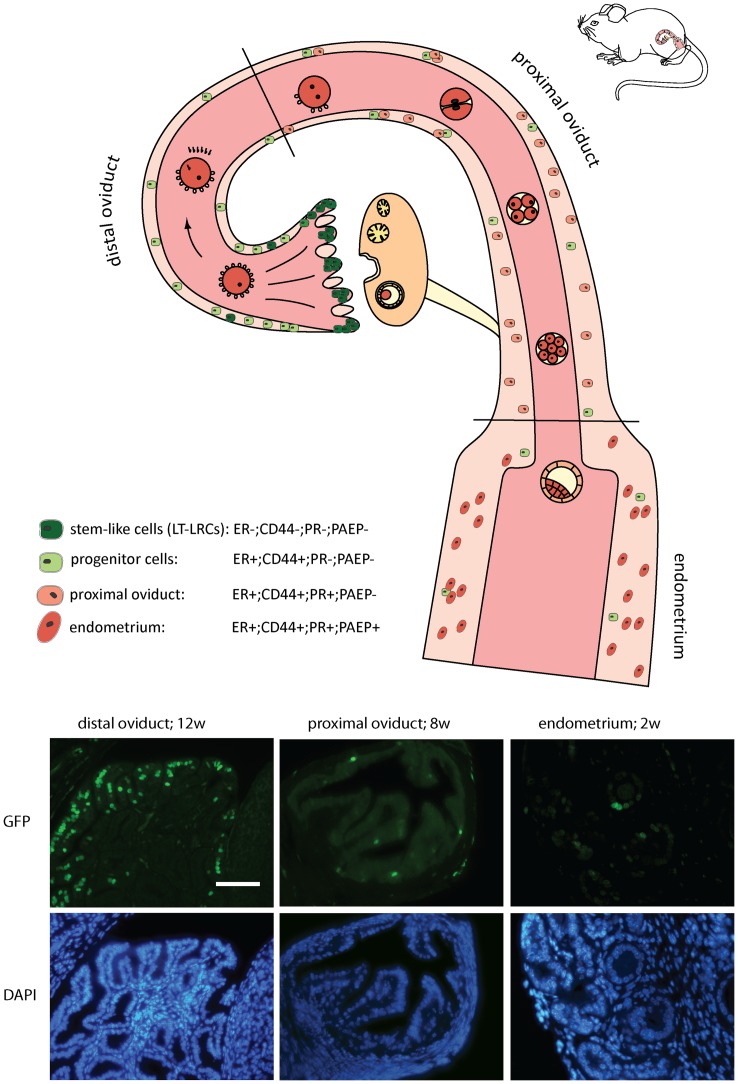
Working Hypothesis. Stem-like LT-LRCs from the distal oviduct (ERα−, PR−, CD44−, and PAEP−) may give rise to progenitor cells (ERα+, PR−, CD44+, and PAEP−) for the epithelial lining of the proximal oviduct (ERα+, PR+, CD44+, and PAEP−) and endometrium (ERα+, PR+, CD44+, and PAEP+). The bottom panels show (LT)-LRCs in the distal oviduct (12 weeks of chase), proximal oviduct (8 weeks of chase) and endometrium (2 weeks of chase).

Hence, upon differentiation of spheroids originating from LT-LRCs, the resulting organoids were found to acquire expression characteristic of cell lineages of the proximal oviduct and endometrium. Based on these results, an hypothetical scenario can be envisaged where stem-like LT-LRCs from the distal oviduct (ERα−, PR−, CD44−, and PAEP−) give rise to progenitor cells (ERα+, PR−, CD44+, and PAEP−) for the epithelial lining of the proximal oviduct (ERα+, PR+, CD44+, and PAEP−) and, possibly, of the endometrium (ERα+, PR+, CD44+, and PAEP+) (Figure 6). However, this model implies extensive migration of stem and progenitor cells from the distal oviduct to the endometrium and only lineage-tracing experiments can deliver definitive evidence for this hypothesis. To this aim, unique markers of LRCs need to be identified to then allow the generation of tracer mouse model by knock-in or transgenic technologies. Notably, the FOXJ1 gene has been shown to be expressed in ciliated cells of the distal oviduct [27] and as such may represent an interesting candidate for future lineage tracing studies. Moreover, specific markers are available to validate differentiation of LRC-derived cells such as PAEP for the epithelial cells in the endometrium and oviduct [28], and cytokeratin 4 for squamous epithelium of the cervix and upper vagina [29].

The finding of quiescent, stem-like cells in the distal female reproductive tract could also be relevant for the ongoing discussion on the origin of ovarian cancer [30], [31], [32]. As discussed below, there are good indications that some ovarian cancer subtypes do not originate from ovarian surface epithelial cells, but from cells in the distal oviduct. The following observations corroborate this hypothesis: (i) reviewing prophylactically removed adnexa from women with a *BRCA1/2* mutation showed a high incidence of serous tubal intraepithelial carcinomas in the distal oviduct and fimbriae [33], [34], [35]. (ii) The three most prevalent ovarian cancer subtypes are morphologically reminiscent of different Müllerian duct-derived structures [36]). (iii) Well-established ovarian cancer biomarkers such as CA125 [37], HE4 [38] and PAX2 [39], are proteins expressed by endometrial and tubal epithelial cells though not in ovarian surface epithelium [37], [38], [39]. In view of our findings, an admittedly speculative though stimulating hypothesis can be formulated. Repeated physical and biochemical insults, such as caused by inflammatory cytokines and reactive oxygen species present during ovulation might trigger cell cycle entrance and expansion in the otherwise quiescent LT-LRCs in the distal oviduct. Expansion of the LT-LCR compartment upon tissue injury, in combination with their differentiation potential, would make these cells candidate cells of origin of distinct ovarian cancer subtypes. Moreover, these cells may also be involved in the development of endometrial benign and malignant aberrations such as endometrial hyperplasia, endometriosis and endometrial cancer.

In summary, we show that quiescent cells located in the distal oviduct including the fimbrial region have stem-like properties and can differentiate into distinct cell lineages resembling endometrium, proximal and distal oviduct. Future lineage-tracing studies will be needed to elucidate the role played by these cells in homeostasis, tissue injury and cancer of the female reproductive tract in the mouse and eventually in man.

## Materials and Methods

### Mice

Transgenic HNRPA2B1/rtTA2S-M2 (rtTA) mice [18] were bred with tetO-HIST1H2BJ/GFP (H2BGFP) mice [1] (kindly provided by Elaine Fuchs, New York). Young adult (8–12 weeks of age) compound heterozygous rtTA/H2BGFP transgenic female mice and control littermates were administered doxycycline (Sigma, Zwijndrecht, the Netherlands) at 2 mg/ml in 5% sucrose containing drinking water for 7 days (pulse). GFP expression in mice was analyzed from 0 till 47 weeks after pulse. This study was carried out in strict accordance with the recommendations in the Guide for the Care and Use of Laboratory Animals of the National Institutes of Health. The protocol was approved by the Committee on the Ethics of Animal Experiments of the Erasmus Medical Center (DEC permit numbers EUR1494 and EUR1785). All efforts were made to minimize suffering.

### Flowcytometry

The oviducts of mice chased for 12 weeks were mechanically (Stomacher® 80 microBiomaster, Seward Limited, West Sussex, UK) and enzymatically dissociated in medium containing 3 mg/ml collagenase (Sigma) and 50 µg/ml DNAse (Sigma) at 37°C, in a 5% CO_2_ incubator for 30 minutes to obtain a single cell suspension. To remove debris, the cell suspension was washed in PBS and passed through a 40 µm cell strainer (BD). To exclude non-epithelial cells from analysis, cells were stained for different lineage markers with biotinylated primary antibodies directed against CD31, CD45 and TER-119, and strepavidin-PerCP-Cy5.5 as a secondary reagent. Cells were incubated with 0.2 μg/ml Hoechst-33342 in PBS for 5 minutes to discriminate live from dead cells. Live cells were sorted as singlets for high (GFP^+^) or very low GFP expression (GFP-negative) by using a FACSAria (BD) flow sorter and plated as single cells in 3D conditions (Matrigel)

### Antibodies and chemicals

Primary antibodies were: biotinylated anti-mouse CD31 (BD, Breda, the Netherlands), biotinylated anti-mouse CD45 (BD), biotinylated anti-mouse TER-119 (BD), anti-CD44 (PE) (BD), rabbit anti-GFP (Invitrogen, Breda, the Netherlands), mouse anti-GFP (Roche, Woerden, the Netherlands), anti-PR (DAKO, Enschede, the Netherlands), anti-β-catenin (Epitomics, Burlingame, USA), anti-ERalpha (Millipore, Amsterdam, the Netherlands), anti-Keratin 8 (Covance, Uden, the Netherlands), anti- Ki67 (Monosan, Uden, the Netherlands), anti-PAEP (Sigma).

Second antibodies were: rabbit/mouse EnVision (DAKO), anti-rabbit immunoglobulins/biotinylated (DAKO), anti-mouse immunoglobulins/biotinylated (DAKO), anti-rat immunoglobulins/biotinylated (DAKO), anti-goat immunoglobulins/biotinylated (DAKO), anti-rat Alexa Fluor 633 (Invitrogen), anti-rat Alexa Fluor Cy3 (Invitrogen), anti-mouse Alexa Fluor 488, 594 (Invitrogen), anti-rabbit Alexa fluor 594 (Invitrogen), fluorescent anti-rabbit (Invitrogen), Strepavidin-PerCP-Cy5.5 (BD), Streptavidin peroxidase (BioGenex, Duiven, the Netherlands). For nuclear staining, the following were employed: Hoechst 33342, 34580 (Invitrogen), DAPI (Invitrogen), DRAQ5 (Biostatus, Leicestershire, UK).

Employed chemicals and biologicals included: phosphate-buffered saline (PBS) (Invitrogen), matrigel matrix (BD), DMEM/F12 (Invitrogen), antibiotic/antimycotic (Invitrogen), gentamycin (invitrogen), recombinant mouse EGF (Invitrogen), recombinant mouse bFGF (Invitrogen), B27 (Invitrogen), DNAse (Sigma), collagenase (Sigma), TrypLE express (Invitrogen), Triton (Sigma), formaldehyde (Merck, Schiphol-Rijk, the Netherlands), Select Agar (Invitrogen).

### *In vitro* cell culture and differentiation assay

After sorting, the cells were washed in cold PBS, re-suspended in matrigel, seeded in 24-well plates (Corning B.V, Amsterdam, the Netherlands), and subsequently covered with culture medium. The culture medium consisted of DMEM/F12 supplemented with 1% v/v B27, 20ng/ml bFGF, 20ng/ml EGF, 100μg/ml gentamycin in the presence of antibiotics and antimycotics. The medium was refreshed every two days.

For the single cell self-renewal assay, FACS sorted GFP+ and GFP-negative cells were allowed to form spheroids for 20 days. Subsequently, the spheroids were washed in cold culture medium and digested in TrypLE express medium for 5 minutes at 37°C, in a 5% CO2 incubator. The resulting single cell suspension was microscopically evaluated, washed in culture medium, resuspended in Matrigel, seeded in a 24-well plate, covered with culture medium, and allowed to form spheroids again. After 20 days this protocol was repeated to again obtain single cells which formed spheroids in culture.

In order to obtain detailed microscopic images of the spheroids, 1 nm doxycycline was used to induce GFP expression in all cells and facilitate their visualization. Images were captured with a confocal microscope LSM510 (Zeiss, Göttingen, Germany). Three-dimensional reconstruction was performed using the LSM510 software (Zeiss) and Amira (Visage, Berlin, Germany) software packages.

For the differentiation assay, spheroids cultured for five weeks were employed. The spheroids were re-suspended in cold culture medium and plated in tissue culture coated 24-well plates. After 4 hours of culture at 5% CO_2_ at 37°C, the medium was carefully removed, and a drop of Matrigel was placed on the top of the spheroids. Subsequently, culture medium supplemented with 10% FCS was added to the Matrigel covering the spheroids to stimulate differentiation. Morphology changes were recorded by confocal microscopy.

### Histology and image analysis

Histological analyses were performed on whole mount spheroids and paraffin-embedded sections of spheroids and mouse tissues. For whole mount staining, the spheroids growing in Matrigel were fixed and permeabilized with 4% formaldyhyde and 1% Triton for 1.5 hours at room temperature, and incubated for 30 minutes with blocking solution (3% BSA; 0.1% Triton). Primary antibodies diluted in blocking solution were then incubated o/n, followed by incubation with the appropriate secondary antibody and subsequent substrate incubation.

To embed morphologically intact spheroids in paraffin, they were first fixed in 4% formaldehyde at 37°C for 2 hours before being transferred to fresh 4% formaldehyde at 4°C overnight. Subsequently, fixed spheroids were transferred into 5% Select Agar which was embedded in paraffin to support sectioning.

Immunohistochemical staining for ER, PR, KI67, CD44, GFP, PAEP, CK8 and beta-catenin was performed as described before [40]. For the double fluorescent staining of ER, PR, KI67, CD44 and GFP, the primary antibodies were added simultaneously. The second antibodies were chosen based on the primary antibodies. Nuclei were stained with Hoechst 34580, DAPI or DRAQ5.

The images were acquired by using Axioplan 2 (Zeiss), Olympus BX41 (Olympus, Hamburg, Germany) and Nanozoomer Digital Pathology (NDP) (Hamamatsu Photonics Deutschland GmbH, Herrsching, Germany).

## Supporting Information

Figure S1 **Pulse-chase experiment using the doxycycline-inducible H2B-GFP system focusing on the endometrium.** Detection of GFP using a fluorescent microscope is shown in A. Details show a strong GFP signal in epithelial cells and a weaker signal in stromal and myometrial cells. Immunohistochemistry for GFP (B+C) was used to detect LRCs in the luminal epithelium (B), or glandular epithelium and stroma (C) in pulsed mice (0 weeks, 0w), and in mice chased for 1 (1w), 2 (2w), 4 (4w), 8 (8w) and 12 weeks. Black arrows point to LRCs in the glandular epithelium at 2 weeks and in the stroma at 4 and 8 weeks.
(PDF)

Figure S2 **Pulse-chase experiment using the doxycycline-inducible H2B-GFP system focusing on GFP signaling in the entire female reproductive tract.** After treatment with doxycycline this mouse was chased for 12 weeks before sacrifice. Staining was performed for GFP and in A, a low magnification overview of uterus, oviduct and ovary is shown. Details are shown in B (LT-LRCs in the distal oviduct), c (proximal oviduct) and D (endometrium).
(PDF)

Figure S3 **Progesterone receptor expression in the female reproductive tract of a 12 week chased animal.** Staining was performed for PR and in A, a low magnification overview of uterus, oviduct and ovary is shown. Details are shown in B (fimbrial region of the distal oviduct), c (proximal oviduct) and D (endometrium).
(PDF)

Figure S4 **Ki67 staining in the fimbrial region of the distal oviduct** (**A**) **and a developing ovarian follicle** (**B**)**.** Yellow arrows indicate rare dividing cells in the fimbrial region of the distal oviduct. Rapidly dividing granulose cells of a developing ovarian follicle are used as a positive control for staining.
(PDF)

Figure S5 **Expression of epithelial markers β-catenin** (**A and B**) **and cytokeratin 8** (**CK8, C and D**) **in the fimbrial region of the distal oviduct** (**A and C**) **and in early spheroids** (**B and D**)**.** Oviducts from 12 week chased mice were dissected for immunohistochemistry and for single cell digestion. The single cell digest was FACSorted for GFP+ cells, which were allowed to form spheroids for 5 days. Two markers, β-catenin (B, green) and CK8 (D, green), were used to stain complete spheroids. The nuclei were contra-stained with DRAQ5 (red). The fluorescent images were captured using confocal microscopy.
(PDF)

Movie S1 **3D reconstruction of confocal microscopy images of a LRC-derived spheroid cultured in the presence of doxycycline to re-induce H2B-GFP expression and allow visualization of every cell.**
(AVI)
