# Supplementary figures and images for: Identification of Quiescent, Stem-Like Cells in the Distal Female Reproductive Tract
Source: PLoS One. 2012 Jul 24;7(7):e40691. doi: 10.1371/journal.pone.0040691 (PMC3404087; doi:10.1371/journal.pone.0040691)

# Supplementary Figure 1

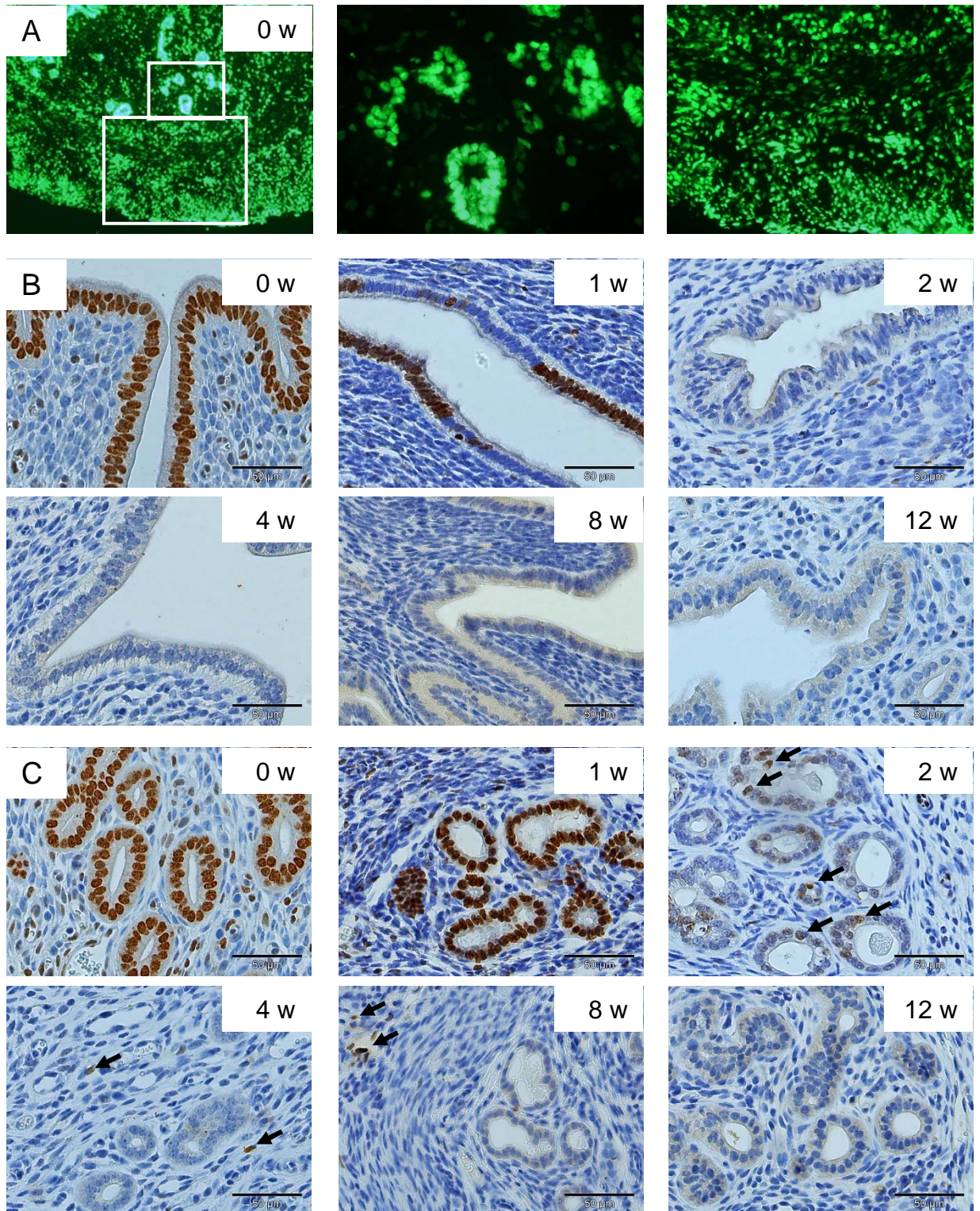

Supplement: Figure S1 — Pulse-chase experiment using the doxycycline-inducible H2B-GFP system focusing on the endometrium. Detection of GFP using a fluorescent microscope is shown in A. Details show a strong GFP signal in epithelial cells and a weaker signal in stromal and myometrial cells. Immunohistochemistry for GFP (B+C) was used to detect LRCs in the luminal epithelium (B), or glandular epithelium and stroma (C) in pulsed mice (0 weeks, 0w), and in mice chased for 1 (1w), 2 (2w), 4 (4w), 8 (8w) and 12 weeks. Black arrows point to LRCs in the glandular epithelium at 2 weeks and in the stroma at 4 and 8 weeks. (PDF) [file pone.0040691.s001.pdf]

# Supplementary Figure 2

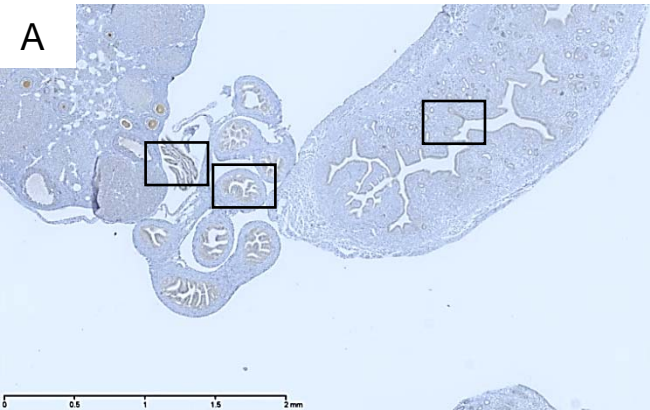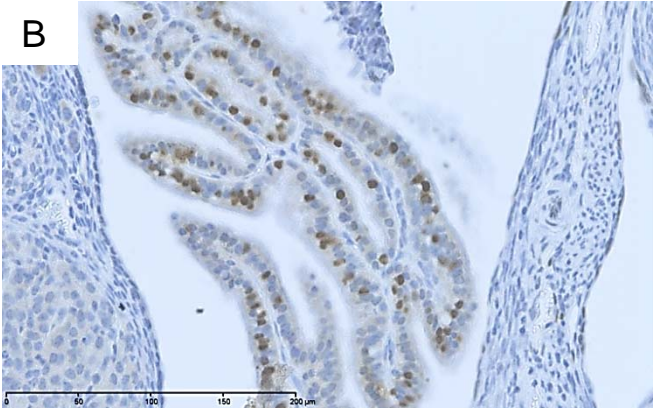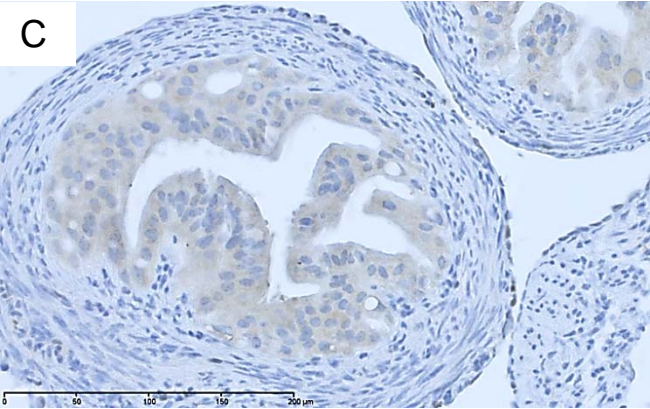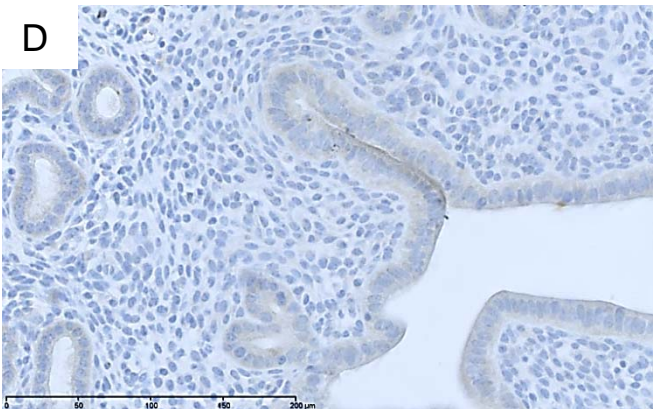

Supplement: Figure S2 — Pulse-chase experiment using the doxycycline-inducible H2B-GFP system focusing on GFP signaling in the entire female reproductive tract. After treatment with doxycycline this mouse was chased for 12 weeks before sacrifice. Staining was performed for GFP and in A, a low magnification overview of uterus, oviduct and ovary is shown. Details are shown in B (LT-LRCs in the distal oviduct), c (proximal oviduct) and D (endometrium). (PDF) [file pone.0040691.s002.pdf]

# Supplementary Figure 3

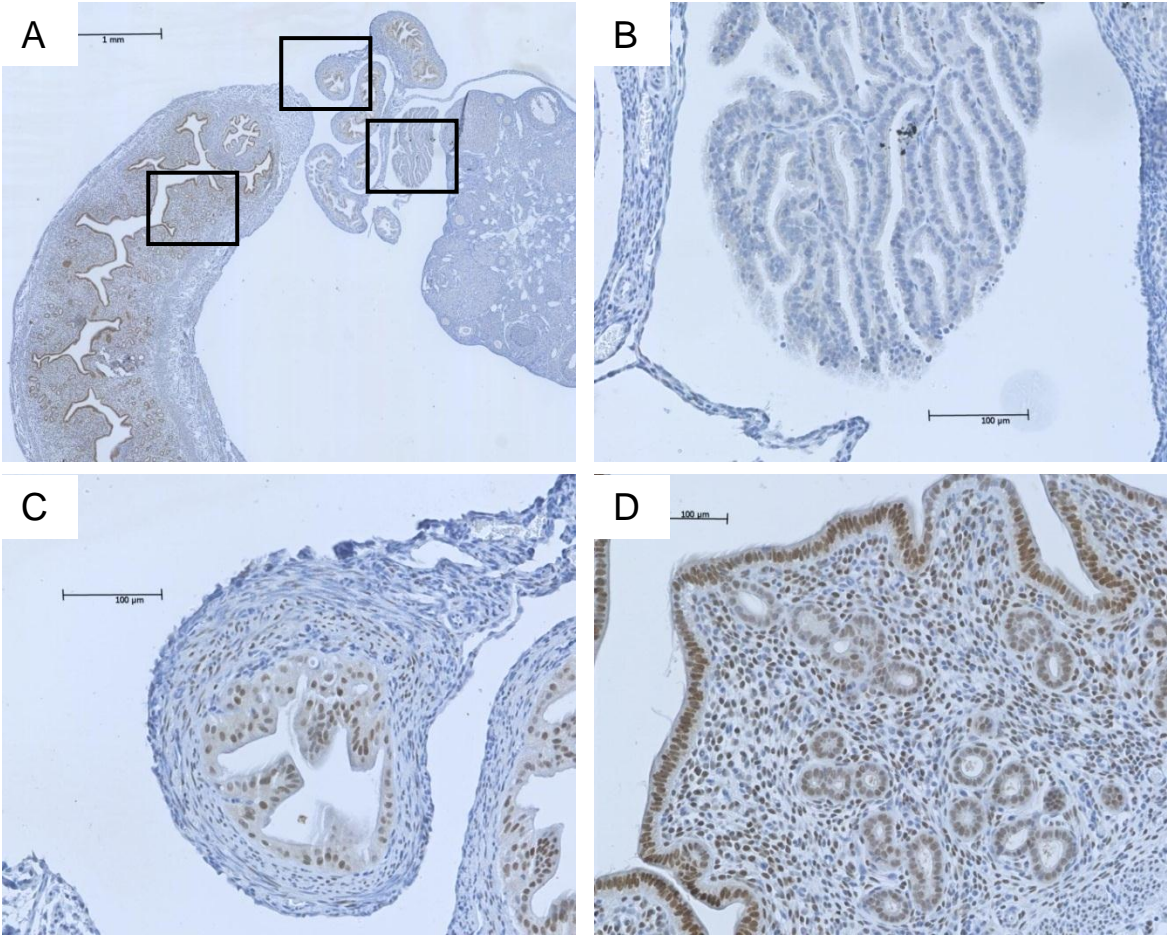

Supplement: Figure S3 — Progesterone receptor expression in the female reproductive tract of a 12 week chased animal. Staining was performed for PR and in A, a low magnification overview of uterus, oviduct and ovary is shown. Details are shown in B (fimbrial region of the distal oviduct), c (proximal oviduct) and D (endometrium). (PDF) [file pone.0040691.s003.pdf]

## Supplementary Figure 4

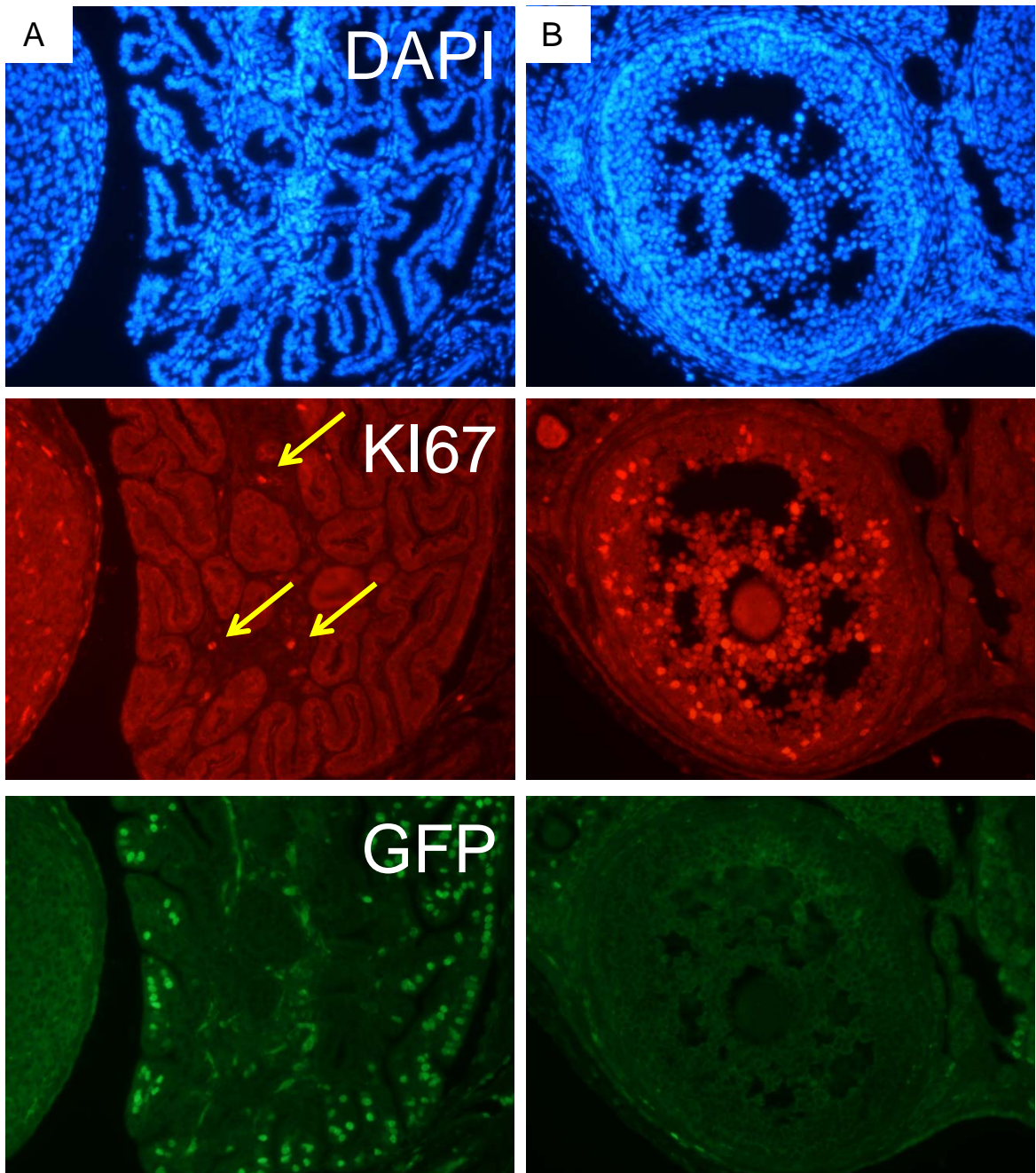

Supplement: Figure S4 — Ki67 staining in the fimbrial region of the distal oviduct (A) and a developing ovarian follicle (B). Yellow arrows indicate rare dividing cells in the fimbrial region of the distal oviduct. Rapidly dividing granulose cells of a developing ovarian follicle are used as a positive control for staining. (PDF) [file pone.0040691.s004.pdf]

# Supplementary Figure 5

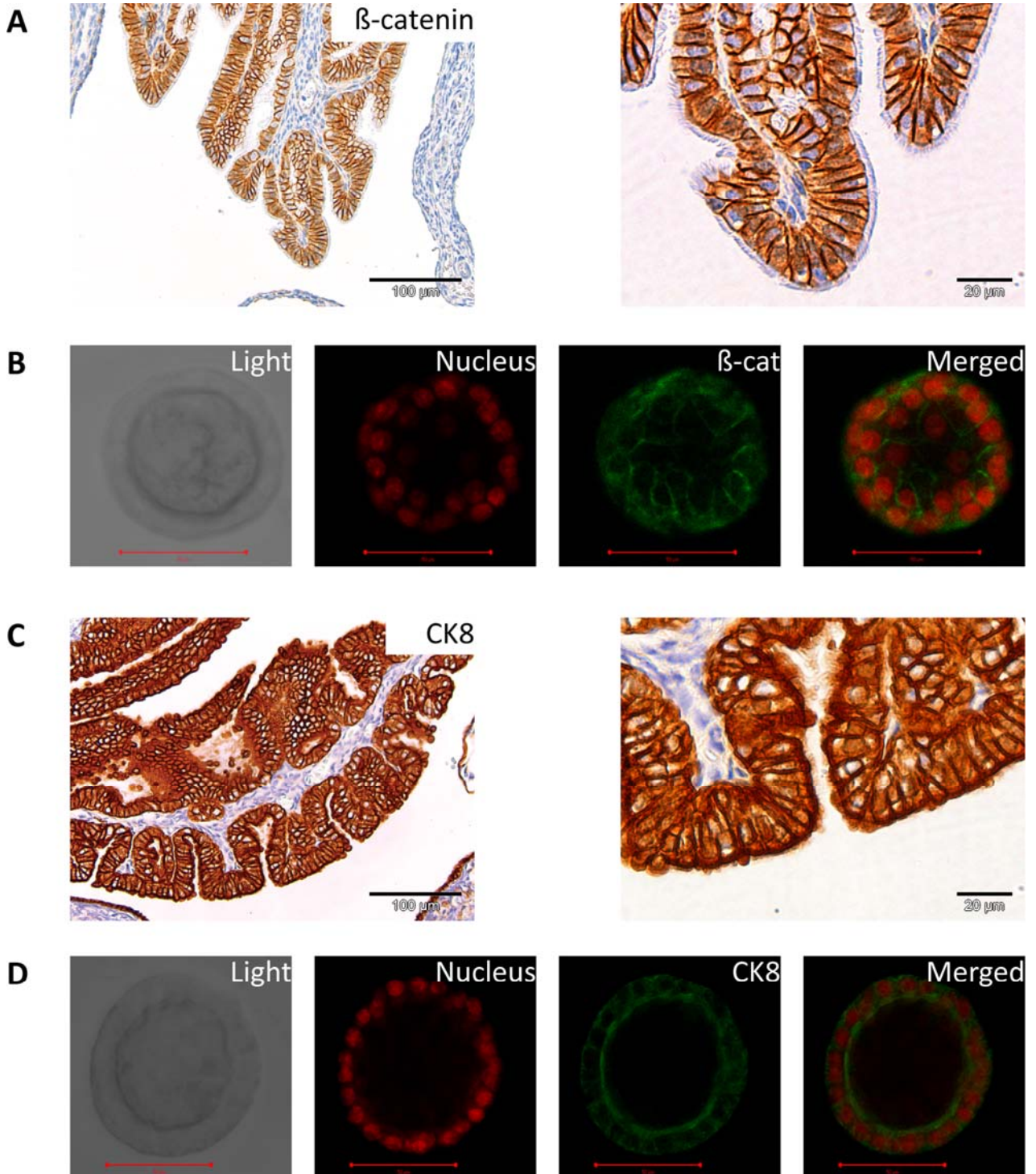

Supplement: Figure S5 — Expression of epithelial markers β-catenin (A and B) and cytokeratin 8 (CK8, C and D) in the fimbrial region of the distal oviduct (A and C) and in early spheroids (B and D). Oviducts from 12 week chased mice were dissected for immunohistochemistry and for single cell digestion. The single cell digest was FACSorted for GFP+ cells, which were allowed to form spheroids for 5 days. Two markers, β-catenin (B, green) and CK8 (D, green), were used to stain complete spheroids. The nuclei were contra-stained with DRAQ5 (red). The fluorescent images were captured using confocal microscopy. (PDF) [file pone.0040691.s005.pdf]
